# Towards two-dimensional room temperature multiferroics

**DOI:** 10.1093/nsr/nwaa258

**Published:** 2020-10-17

**Authors:** Hongjun Xiang

**Affiliations:** Key Laboratory of Computational Physical Sciences (Ministry of Education), State Key Laboratory of Surface Physics, and Department of Physics, Fudan University, China; Collaborative Innovation Center of Advanced Microstructures, China; Shanghai Qi Zhi Institute, China

Multiferroic materials with coupled ferroelectricity (FE) and magnetism have long been sought for novel memory devices [1–3]. The co-existence of FE and magnetism is rare in nature, which can be attributed to their mutual exclusive origins (empty d shell for conventional ferroelectric order and partially filled d shell for magnetic order). Moreover, magnetoelectric (ME) coupling is weak in type-I multiferroics with FE and magnetism arising respectively from different mechanisms, while for type-II multiferroics with FE induced by magnetic ordering, their low spin-driven ferroelectric polarizations (mostly <0.01 C/m^2^) and Curie temperature (mostly <150 K) hinder their practical applications [4,5]. To date, almost all synthesized magnetoelectric multiferroics have been three-dimensional.

In a recent work, Zhong *et al.* [6] instead focused on 2D ferroelectrics [7] and predicted a room temperature multiferroic with a desirable co-existence of ferromagnetism (FM) and FE and strong magnetoelectric coupling. To be more specific, they investigated 2D thin-layer CuCrX_2_ (X = S or Se). The Curie temperatures of FM and FE were both above room temperature, where the FM is stabilized by enhanced carrier density and polarization-driven orbital shifting. Moreover, the gradient of interlayer coupling parameter between adjacent layers gave rise to diversified types of magnetoelectric layers of different thicknesses. For example, tri-layer Cu-intercalated CrS_2_, denoted as Cu_2_(CrS_2_)_3_, is ferroelectric in-plane while ferrimagnetic vertically as shown in Fig. 1(a), with a net magnetization of 2.62 μ_B_/f.u. For the ground state with polarization downwards, the middle layer is antiferromagnetically coupled with the down layer while ferromagnetically coupled with the top layer; when the polarization is upwards, the magnetization of the middle layer will be reversed, ferromagnetically coupled with the down layer while antiferromagnetically coupled with the top layer. Hence FE switching should enable a 180-degree reversal of a considerable magnetization of 2.62 μ_B_/f.u. The ground state for four-layer Cu-intercalated CrS_2_ denoted as Cu_3_(CrS_2_)_4_ is shown in Fig. 1(b), where the upper two layers are ferromagnetically coupled while antiferromagnetically coupled with the two layers downwards. The net magnetization of 0.35 μ_B_/f.u., which is much reduced, can also be reversed via polarization switching. The swapping of spin-up and spin-down channel in band structures during FE switching may result in a new type of ‘electrical writing + magnetic reading’ memory architecture.

**Figure 1.**
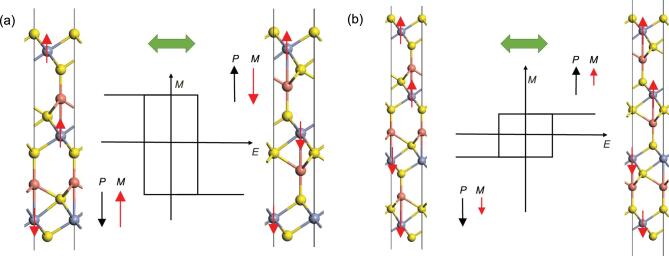
Spin configurations and multiferroic switching for (a) Cu_2_(CrS_2_)_3_ and (b) Cu_3_(CrS_2_)_4_ thin films. Black and red arrows denote the directions of polarization and magnetization, respectively. Adapted from Fig. 4 of Ref. [6].

The work by Zhong *et al.* [6] not only paves a new way to realize a room temperature ferromagnetic-ferroelectric multiferroic with strong magnetoelectric coupling [5,8,9], but may also stimulate more studies on multiferroicity in 2D systems. It remains to be seen whether the 2D multiferroic material or concept conveyed in this study can be experimentally confirmed or whether the predicted ME coupling can be confirmed in a more direct simulation of the FE switching process.

## FUNDING

This work was supported by the National Natural Science Foundation of China (11825403 and 11991061), the Program for Professor of Special Appointment (Eastern Scholar) and the Qing Nian Ba Jian Program.

***Conflict of interest statement*.** None declared.
